# Which symptoms are the psychopathological core affecting the manifestation of pseudo-cardiac symptoms and poor sleep quality in young adults? Symptoms of personality disorders versus clinical disorders

**DOI:** 10.3389/fpsyg.2022.1011737

**Published:** 2022-12-09

**Authors:** Mostafa Bahremand, Saeid Komasi

**Affiliations:** ^1^Department of Cardiology, Faculty of Medicine, Kermanshah University of Medical Sciences, Kermanshah, Iran; ^2^Department of Neuroscience and Psychopathology Research, Mind GPS Institute, Kermanshah, Iran

**Keywords:** mental symptoms, personality disorder, pseudo-cardiac symptoms, psychological assessment, sleep disturbance

## Abstract

**Background:**

Diagnosing and identifying the psychological origin of pseudo-cardiac symptoms and comorbid conditions such as poor sleep quality is very difficult due to its extensive and complex nature. The present study was conducted to determine the contribution of symptoms of personality disorders (PDs) and clinical disorders (CDs; i.e., psychological symptoms measured using the Symptom Checklist-90) to the manifestation of pseudo-cardiac symptoms and poor sleep quality.

**Methods:**

Subjects in this cross-sectional study were 953 (64.3% female; 28.8 ± 6.2 years) community samples in the west of Iran who were selected by convenience sampling. After applying the inclusion criteria, data were collected using the Symptom Checklist-90 (SCL-90-R), the Personality Diagnostic Questionnaire (PDQ-4), and the Scale for Pseudo-Cardiac Symptoms and Poor Sleep Quality (SPSQ). Pearson correlations, factor analytical techniques, and hierarchical regression models were used to examine associations between symptoms of PDs/CDs and outcome factors.

**Results:**

Factor analytical techniques confirmed both the integrated structure of symptoms of PDs and CDs. Both pseudo-cardiac symptoms and poor sleep quality were more strongly associated with symptoms of CDs than PDs. The results of the hierarchical analysis show that the CDs factor alone could explain the total variance of both pseudo-cardiac symptoms (change in R^2^ = 0.215 vs. 0.009; *p* < 0.001) and poor sleep quality (change in R^2^ = 0.221 vs. 0.001; *p* < 0.001).

**Conclusion:**

The different capabilities of two unique factors for the symptoms of PDs and CDs were confirmed by factor analytical methods and regression analysis techniques. Although each of the symptoms of PDs and CDs independently contributes to the manifestation of pseudo-cardiac symptoms and poor sleep quality, the CDs factor is the psychopathological core.

## Introduction

Cardiovascular diseases (CVDs) are the first cause of death worldwide, killing more than 19 million people annually (Tsao et al., 2022). Different age and gender groups around the world generally underestimate the risk of CVDs despite adequate knowledge about chest pain and cardiac symptoms (Helou et al., 2018; Koohi and Khalili, 2020). High health literacy and knowledge make different age and sex groups recognize such symptoms well and immediately seek treatment to reduce the possible risk of sudden death (Magnani et al., 2018). Recent studies have reported that knowledge of cardiac symptoms and fear of cardiac death lead to an immediate referral to emergency rooms and medical care centers (Magnani et al., 2018; Tremblay et al., 2018). However, this situation also applies to pseudo-cardiac symptoms without the risk of death (Tremblay et al., 2018). These symptoms are also associated with serious problems such as occupational problems, reduced quality of life, poor sleep quality, and sleep disorders (Pedrosa et al., 2010; Félin-Germain et al., 2018; Meresh et al., 2018). Sleep disorders and poor sleep quality in particular can be both a risk factor and a consequence of CVDs (Kwok et al., 2018; Getahun et al., 2021). Sleep quality is defined as a person’s satisfaction with various aspects of the sleep experience and it includes the dimensions of sleep efficiency, latency and duration, and wake after sleep onset (Nelson et al., 2022). In recent years, the benefits of good sleep quality on physical, physiological, and psychological health have been noted in numerous studies (Tahmasian et al., 2020; Clement-Carbonell et al., 2021).

Unlike cardiac symptoms such as chest pain and tachycardia, pseudo-cardiac symptoms do not have a cardiovascular origin and are usually not fatal. These symptoms are common in about 13% of the general population (Ford et al., 2011). Pseudo-cardiac symptoms usually have a gastrointestinal, pulmonary, musculoskeletal, neurological, or psychological origin (Fass and Achem, 2011). Although patients usually refer to medical care centers due to the fear of CVDs and the risk of death due to it (Tremblay et al., 2018), a set of services such as medical interviews, laboratory tests, and medical imaging techniques strongly help to identify the origin of pseudo-cardiac symptoms in initial visits (Sobański et al., 2016; Wertli et al., 2019). If the origin of the symptoms is diagnosed and treated, at this stage, patients usually refrain from future visits. Otherwise, patients frequently refer to medical care centers, which impose a lot of costs on the health systems (Carroll, 2017; Bouck et al., 2019).

Somatic symptoms such as pseudo-cardiac symptoms are usually comorbid with sleep disorders and poor sleep quality. For example, a recent review reported that the prevalence of insomnia in somatoform patients is 20–48% (Ionescu et al., 2021). Another report points to a 44% prevalence of insomnia in people with unexplained chest pain (Belleville et al., 2014). Pseudo-cardiac symptoms can also occur concurrently with other sleep problems such as insufficient sleep duration, more sleep episodes, longer sleep latency, and poor sleep quality (Lewandowski Holley et al., 2017; Frange et al., 2019; Chen et al., 2022). Although both pseudo-cardiac symptoms and poor sleep quality may have a psychological origin (Fass and Achem, 2011; Belleville et al., 2014), identifying the underlying and sustaining psychological components of these problems is very difficult due to their complex and extensive nature (Rief and Broadbent, 2007). Previous studies have pointed to some factors including psychological distress (Mourad et al., 2018), maladaptive and irrational beliefs (Bahremand et al., 2015; Komasi et al., 2016), mental disorders such as acute stress disorder, generalized anxiety disorder, depression, substance abuse, and anorexia (Eken et al., 2010; Belleville et al., 2014; Foldes-Busque et al., 2016; Ionescu et al., 2021), and personality components (García-Campayo et al., 2010; Rezaei et al., 2021).

### The study aims

Although the research literature refers to numerous studies involving psychological factors, personality traits, and mental disorders contributing to pseudo-cardiac symptoms and poor sleep quality (Eken et al., 2010; García-Campayo et al., 2010; Fass and Achem, 2011; Belleville et al., 2014; Bahremand et al., 2015; Foldes-Busque et al., 2016; Komasi et al., 2016; Sobański et al., 2016; Mourad et al., 2018; Ionescu et al., 2021; Rezaei et al., 2021), it seems that the importance of personality disorders (PDs) has been widely ignored. On the other hand, the comparative investigation of the symptoms of PDs and non-PDs or clinical disorders (CDs; i.e., psychological symptoms measured using the Symptom Checklist-90), especially regarding pseudo-cardiac symptoms, has been neglected. In other words, our search was fruitless to find studies that compared the contribution of PDs and CDs to the manifestation of pseudo-cardiac symptoms and comorbid conditions such as poor sleep quality. Although the current dimensional frameworks in psychopathology propose the integration of all mental disorders, including PDs and CDs (Kotov et al., 2017), such a claim cannot be definitively accepted until sufficient empirical evidence is provided (Komasi et al., 2022). Therefore, we sought to answer the question of whether factor analysis supports a one-factor or two-factor structure for the symptoms of PDs and non-PDs. If a separate two-factor structure is proposed, it can be beneficial to study the specific contribution of each of them to the manifestation of somatic symptoms and comorbid conditions such as poor sleep quality. Although previous studies have not shown interest in maladaptive personality traits and PDs, some previous studies have mentioned the role of personality traits and CDs in pseudo-cardiac symptoms and comorbid conditions, especially in the elderly population (Kuijpers et al., 2007; Belleville et al., 2014; Foldes-Busque et al., 2016; Sobański et al., 2016; Jiang et al., 2017; Ionescu et al., 2021; Roohafza et al., 2022). Considering that younger people show more somatization and pseudo-cardiac symptoms than the elderly (Hilderink et al., 2013), younger samples were the target population of our study. Based on these considerations, we proposed two hypotheses: (i) factor analysis supports a two-factor structure for the symptoms of PDs and CDs; (ii) CDs have a better ability than PDs to both the manifestation of pseudo-cardiac symptoms and poor sleep quality.

## Materials and methods

### Design, sampling, and data collection

**Figure 1:**
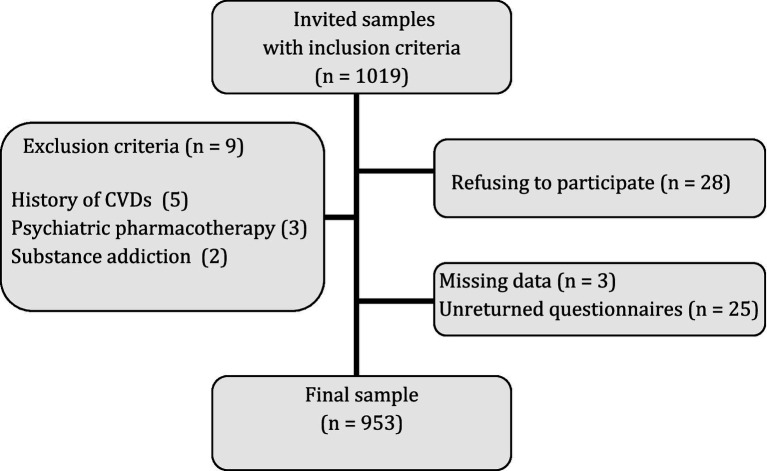
The sampling process.

The current cross-sectional study includes 953 (613 female; 64.3%) community samples from the city of Kermanshah in the west of Iran. The participants were recruited through convenience sampling between August 2020 and May 2021. Data were collected by clinical psychologists of the research team after obtaining informed consent. The inclusion criteria were 18 to 40 years old and fluency in the Farsi language. Circumstances such as a history of CVDs and cerebrovascular diseases, any invasive general surgery or psychiatric pharmacotherapy in the last 14 days, and current substance addiction were the criteria for excluding the samples from the study. Also, outliers and questionnaires with more than 15% of missing data were excluded from the study. Figure 1 shows the sampling process in more detail.

The age means and standard deviation of the subjects were 28.8 ± 6.2 years. The participants were mostly single (58.7%), diploma or college degree (93%), employed, self-employed, housewives, or college students (83.7%), lack of any medical visit or procedure in the last 30 days (94.1%), no history of smoking (83.8%), alcohol abuse (88.9%), and substance abuse (98.1%). To collect data, the study process was first explained to the subjects by two expert clinical psychologists. After obtaining informed consent to participate in the study, the subjects completed the sociodemographic checklist (gender, age, education level, job, marital status, physical illness and treatment, and history of smoking, alcohol abuse, and substance abuse). All participants answered the Revised Form of Symptom Checklist-90 (Derogatis et al., 1976; Derogatis and Unger, 2010; Anisi et al., 2016), the Fourth Edition of the Personality Diagnostic Questionnaire (Bagby and Farvolden, 2004; American Psychiatric Association, 2013; Yousefi et al., 2021), and the Scale for Pseudo-Cardiac Symptoms and Poor Sleep Quality (SPSQ; 11 items).

### Data measurement

#### Revised form of symptom checklist-90

This 90-items checklist was developed to assess the symptoms of mental disorders. The revised format also was prepared by Derogatis et al. (1976) and Derogatis and Unger (2010). Nine clinical dimensions include somatization, obsessive–compulsive disorder, interpersonal sensitivity, depression, anxiety, hostility, phobic anxiety, paranoid ideation, and psychoticism. This checklist also includes seven additional items. The answers to each item are graded based on a five-point Likert scale from zero (no discomfort) to four points (very severe discomfort). The validity and reliability of the original version (Derogatis et al., 1976; Derogatis and Unger, 2010) and the Persian version (Anisi et al., 2016) of SCL-90-R have already been confirmed.

#### Fourth edition of the personality diagnostic questionnaire

PDQ-4 is a 100-item self-report questionnaire that was designed to diagnose symptoms of PDs. This dimensional questionnaire evaluates symptoms of 12 PDs including paranoid, schizoid, schizotypal, antisocial, borderline, narcissistic, histrionic, avoidant, dependent, obsessive–compulsive, depressive, and negativistic (7, 7, 9, 7, 9, 9, 8, 7, 8, 8, 7, and 7 items, respectively) PDs. The answer to the items is yes (score 1) or no (score 0). The validity and reliability of the original version (Bagby and Farvolden, 2004) and the Persian version (Yousefi et al., 2021) of PDQ-4 have already been confirmed. According to the diagnostic categories presented in section II DSM-5 (American Psychiatric Association, 2013), two subscales of depressive and negativistic PDs were excluded from the present study.

#### Scale for pseudo-cardiac symptoms and poor sleep quality

We adapted seven items about cardiac symptoms including palpitations, discomfort in the heart area, and chest pain as well as four items about poor sleep quality from three questionnaires of SCL-90-R (items 12, 39, 44, 64, and 66), the Screening for Somatic Symptom Disorders-7 (SOMS-7; items 6, 24, and 25), and the Patient Health Questionnaire-15 (PHQ-15; items 6, 9, and 15). SOMS-7 is a 47-item questionnaire to evaluate the severity of the somatic signs/symptom during the last 7 days. Each item is scored directly on a 4-point Likert scale from never (score 0) to always (score 3). The scale that was developed by Rief and Hiller (2003) is a valid and reliable questionnaire for Iranian populations (Ebrahimi et al., 2018). Also, the PHQ-15 is designed to measure the severity of somatic symptoms during the past 7 days. Each item on the questionnaire is rated on a three-point scale from zero (not at all) to 2 (a lot). The scale has good reliability and the validity of the PHQ-15 also is acceptable in Iranian samples (Shabbeh et al., 2016). In the results section of the present study, we have reported the results of the Exploratory Factor Analysis (EFA) and Confirmatory Factor Analysis (CFA) as well as Cronbach’s alpha for the SPSQ questionnaire.

### Data analysis

First, seven items related to pseudo-cardiac symptoms (palpitations, discomfort in the heart area, and chest pain) and four items for poor sleep quality were selected from the SOMS-7, PHQ-15, and SCL-90-R. Then, we planned a conjoint EFA with maximum likelihood estimations on all items. These factors were rotated using Varimax rotation. This analysis led to the identification of two factors (criterion variables in the present study), the details of which can be seen in the results section. In the next step, a CFA was performed for the factors identified by CFA. In the nest stage, the means and standard deviations of all predictor variables and the Pearson correlations between all symptoms of SCL-90-R and PDQ-4 were reported. The extremely strong correlation between many of these predictor variables (multi-collinearity) was the next challenge in running the regression techniques. Moreover, previous research suggests that each of these scales has issues related to discriminant validity (Brophy et al., 1988; McCabe and Widiger, 2020). Therefore, to identify latent factors, we conducted another conjoint EFA with maximum likelihood estimations on both sets of scales. To identify the simple structure of these homogeneous variables, these factors were rotated using Promax rotation (Finch, 2006). This analysis led to the identification of two factors including the symptoms of PDs and CDs (predictor variables), the details of which can be seen in the results section. Regarding the sufficiency of the sample size for all factor analyses, the sample size of the present study does not violate previous reports that emphasize the necessity of at least 10 cases for each item, and the subjects-to-variables (STV) ratio should be no lower than five (Bryant and Yarnold, 1995) or at least 100 cases and an STV ratio of no less than five (Suhr, 2006).

We then used hierarchical multiple regression techniques in which the PDs and CDs factors were entered as blocks to predict pseudo-cardiac symptoms and poor sleep quality. For each outcome variable, we entered the PDs scores first in one model and the CDs scores first in another model. We compared the change in R^2^ to determine how much additional variance each model was explaining in the outcome. Tabachnick and Fidell (2013) recommend a formula of N! 50 + 8 m where m is the number of independent variables. So, the sample size was sufficient for all regression analyses. All analyses were performed using the SPSS-20 and AMOS software and *p* ≤ 0.05 was considered the significance level.

## Results

Table 1 shows the conjoint EFA with maximum likelihood estimations using Varimax rotation on 11 items related to pseudo-cardiac symptoms and poor sleep quality on SOMS-7, PHQ-15, and SCL-90-R. KMO (0.871) and Bartlett’s test (4423.020, *p* < 0.001) statistics confirmed the suitability of the data for structure detection. We found two factors with eigenvalues >1 (4.799 and 1.599). These factors could explain 50.1% of the variance. The goodness of fit of the model was confirmed using CFA (GFI = 0.906, CFI = 0.895, PNFI = 0.693). Internal consistency was acceptable for both factors I (α = 0.835) and II (α = 0.783) and the total scale (α = 0.851).

**Table 1:** Rotated factor matrix of several items related to the pseudo-cardiac symptoms and poor sleep quality.

Variables	Factor 1: Pseudo-cardiac symptoms	Factor 2: Poor sleep quality
SOMS-7: Item 6 (Chest pain)	**0.589**	0.188
SOMS-7: Item 24 (Heart palpitations)	**0.696**	0.193
SOMS-7: Item 25 (Heart discomfort)	**0.732**	0.155
PHQ-15: Item 6 (Chest pain)	**0.699**	0.122
PHQ-15: Item 9 (Heart palpitations)	**0.670**	0.205
PHQ-15: Item 15 (Sleep problem)	0.225	**0.639**
SCL-90-R: Item 12 (Chest pain)	**0.575**	0.268
SCL-90-R: Item 39 (Heart palpitations)	**0.604**	0.318
SCL-90-R: Item 44 (Initial insomnia)	0.160	**0.771**
SCL-90-R: Item 64 (Late insomnia)	0.312	**0.433**
SCL-90-R: Item 66 (Low sleep quality)	0.177	**0.888**

All columns contain Pearson correlation coefficients (r) between the variables and the latent factors. Items related to each factor are highlighted. SOMS-7, screening for somatic symptom disorders; PHQ-15, patient health questionnaire; SCL-90-R, revised form of symptom checklist-90. Bold values indicate stronger factor loadings.

The mean, standard deviation, and correlation matrix between the subscale of the SCL-90-R and PDQ-4 can be seen in a Supplementary Table S1. The results of this table suggest a high degree of homogeneity in the patterns across SCL-90-R and PDQ-4 scales. Therefore, we identified latent factors using another conjoint EFA with maximum likelihood estimations on both sets of scales. We rotated these factors with Promax to achieve pattern coefficients.

Table 2 presents the pattern coefficients from a conjoint EFA of SCL-90 and PDQ-4 symptom scales. KMO (0.994) and Bartlett’s test (14563.165, *p* < 0.001) statistics confirmed the suitability of the data for structure detection. We found two factors with eigenvalues >1 (9.198 and 2.844) for the symptoms of CDs and PDs. All scales of SCL-90-R loaded on the first factor, whereas all scales of PDQ-4 loaded on the second. All coefficients were quite strong (> 0.53) and cross-factor coefficients were all weak (| < 0.21|). These factors could explain 59.3% of the variance. Internal consistency was acceptable for both factors I (α = 0.955) and II (α = 0.885) and the total scale (α = 0.922).

**Table 2:** The pattern coefficients from a conjoint exploratory factor analysis of SCL-90 and PDQ-4 symptom scales.

Variables	Factor 1: CDs symptoms	Factor 2: PDs symptoms
SCL-90-R somatization	0.888	−0.107
SCL-90-R OCD	0.900	−0.031
SCL-90-R interpersonal sensitivity	0.904	0.024
SCL-90-R depression	0.924	−0.022
SCL-90-R anxiety	0.954	−0.042
SCL-90-R hostility	0.784	0.055
SCL-90-R phobic anxiety	0.837	−0.041
SCL-90-R paranoid ideation	0.737	0.140
SCL-90-R psychoticism	0.868	0.025
PDQ-4 paranoid	−0.041	0.639
PDQ-4 schizoid	−0.019	0.591
PDQ-4 schizotypal	−0.007	0.683
PDQ-4 antisocial	−0.046	0.690
PDQ-4 borderline	0.207	0.624
PDQ-4 narcissistic	−0.115	0.776
PDQ-4 histrionic	−0.047	0.675
PDQ-4 avoidant	0.139	0.571
PDQ-4 dependent	0.203	0.538
PDQ-4 obsessive–compulsive	−0.034	0.678

All columns contain Pearson correlation coefficients (r) between the variables and the latent factors. CDs, clinical disorders; PDs, personality disorders; OCD, obsessive–compulsive disorder; SCL-90-R, revised Form of symptom checklist-90; PDQ-4, fourth edition of the personality diagnostic questionnaire.

Table 3 shows the results of hierarchical regression models comparing PDs and CDs factors as blocks to predict pseudo-cardiac symptoms and poor sleep quality. In general, PDs and CDs factors together could explain 25.6 and 30.5% of the variance of pseudo-cardiac symptoms and poor sleep quality, respectively. Our main focus is on the relative change in R^2^ values for models with pseudo-cardiac symptoms and poor sleep quality as the dependent variables (DVs). When the CDs factor was entered in the first block, it had an R^2^ of 0.247 (β = 0.491, *p* < 0.001) for pseudo-cardiac symptoms and 0.304 (β = 0.551, *p* < 0.001) for poor sleep quality, whereas when the PDs factor was entered in the first block, it had an R^2^ of 0.041 (β = 0.202, *p* < 0.001) for pseudo-cardiac symptoms and 0.083 (β = 0.288, *p* < 0.001) for poor sleep quality. CDs factor explained more variance above and beyond the PDs factor when predicting pseudo-cardiac symptoms (change in R^2^ = 0.215 vs. 0.009) and poor sleep quality (change in R^2^ = 0.221 vs. 0.001). Overall, these results confirm that both sets of dimensions are relevant to both pseudo-cardiac symptoms and poor sleep quality.

**Table 3:** The hierarchical regression models comparing PDs and CDs factors as blocks to predict pseudo-cardiac symptoms and poor sleep quality.

Variables	Block 1 R^2^: validity	Beta	*p*	Block 2 R^2^: incremental validity	Beta	*p*	Cumulative R^2^
Pseudo-cardiac symptoms							0.256
CDs factor	0.247	0.491	0.001	0.215	0.563	0.001	
PDs factor	0.041	0.202	0.001	0.009	−0.117	0.001	
Poor sleep quality							0.305
CDs factor	0.304	0.551	0.001	0.221	0.571	0.001	
PDs factor	0.083	0.288	0.001	0.001	−0.035	0.290	

CDs, clinical disorders; PDs, personality disorders.

## Discussion

The main purpose of this study was to determine the contribution of symptoms of PDs and CDs to both the manifestation of pseudo-cardiac symptoms and poor sleep quality. We hypothesized that factor analysis supports a two-factor structure for the symptoms of PDs and CDs. The results of the present study supported this hypothesis and data analysis using factor analytical techniques showed that the symptoms of PDs and CDs are two independent factors. Although this finding is inconsistent with the current literature in psychopathology (Kotov et al., 2017; Gluschkoff et al., 2019; McCabe et al., 2022), it is consistent with the findings of some studies in Western and non-Western samples (Bachrach et al., 2012; Komasi et al., 2022). The present results suggest that, although it is possible to synthesize psychopathological differences into a single framework (Kotov et al., 2017), there is nevertheless a difference between PDs and CDs. We also observed strong intercorrelations between all symptoms of PDs and CDs. Although this may be due to the high overlap of individual differences factors and the integrated structure of psychopathology (Gluschkoff et al., 2019), discriminant validity issues in particular measures should not be ignored (Brophy et al., 1988; McCabe and Widiger, 2020). The strong intercorrelations may also be the result of comorbidity between the symptoms of PDs and CDs (Bachrach et al., 2012). Previous evidence supports the role of genetics and similar patterns of stability in both PDs and CDs (Coleman et al., 2020; Hopwood et al., 2020). Despite these similarities, our results showed that the covariance of symptoms is different between these disorders. That is, the increase in the scores of one factor does not coincide with the increase in the scores of another factor (cross-factor coefficients were all weak). The hierarchical regression techniques used by us also showed that these two independent factors are differentially associated with pseudo-cardiac symptoms and poor sleep quality.

Our main question is, what is the contribution of symptoms of PDs and CDs in the manifestation of pseudo-cardiac symptoms and poor sleep quality? Considering that somatic and sleep symptoms are strongly influenced by anxiety and mood symptoms (Eken et al., 2010; Belleville et al., 2014; Foldes-Busque et al., 2016; Mourad et al., 2018; Tahmasian et al., 2020), we hypothesized that CDs have a better ability than PDs to explain both pseudo-cardiac symptoms and poor sleep quality. Our results supported this hypothesis and it turned out that CDs have a better ability than PDs to both the manifestation of pseudo-cardiac symptoms and poor sleep quality. Surprisingly, when the CDs factor was entered in the first block and the PDs factor in the second block, the residual contribution of the PDs factor to explaining the variance of the criterion variable was less than 1 %. Although our search failed to find results from similar studies, previous studies have highlighted the importance of psychological distress, symptoms of anxiety and depression, and clinical disorders such as acute stress disorder, panic disorder, and generalized anxiety disorder (Eken et al., 2010; Foldes-Busque et al., 2016; Mourad et al., 2018; Heppell et al., 2021; Ionescu et al., 2021; Hamel et al., 2022). We assume that maladaptive personality traits are a stable part of the person that has no insight into them. Conversely, symptoms of clinical disorders such as panic attacks and major depressive disorder may be less persistent, and their sudden onset is usually associated with a reasonable level of insight (He et al., 2018). Also, some of the symptoms of CDs such as heart palpitations in panic attacks are quite similar to real heart symptoms. Also, the physiological mechanisms involved in both CDs such as anxiety/depressive symptoms and somatization can be discussed (Rief and Broadbent, 2007). Another possible explanation is the relationship between negative affect or emotions and somatic symptoms (Schwarz et al., 2017). The results of a recent systematic review have confirmed the association between emotion dysregulation and somatic symptoms (Okur Güney et al., 2019). According to the most practical current psychopathology framework (Kotov et al., 2017), CDs factor is more related to the internalizing spectrum and super-spectrum of emotional dysfunction than the PDs factor.

We also intended to determine the contribution of symptoms of PDs and CDs to the manifestation of poor sleep quality. As mentioned earlier, the present results showed that CDs have a better ability than PDs to explain poor sleep quality. Although our search failed to find results from similar studies, this finding was expected. Sleep disorders and poor sleep quality are not only associated with cardiac symptoms (Kwok et al., 2018; Lao et al., 2018; Getahun et al., 2021), but previous reports have confirmed their relationship with psychosomatic and pseudo-cardiac symptoms (Fass and Achem, 2011; Jiang et al., 2017; Ionescu et al., 2021). Therefore, part of the sleep problems may be caused by somatic symptoms. Somatic symptoms are strongly related to health-focused ruminations and illness anxiety (Sansone and Sansone, 2012). Another part can be the result of other predisposing or consequent mental disorders for pseudo-cardiac symptoms (Baglioni et al., 2016). This claim was confirmed by the findings of the present study. Because our results indicated that symptoms of CDs explain poor sleep quality much better than symptoms of PDs. Surprisingly, when the CDs factor was entered in the first block and the PDs factor in the second block, the residual contribution of the PDs factor to explaining the variance was nearly 0 %. Although the quality of sleep in people with PDs is poor compared to those without disorders (Zhang and Lu, 2013), it seems that symptoms of CDs related to the internalizing spectrum have a greater contribution (Kotov et al., 2017).

## Limitations and recommendations

To our knowledge, the present study is pioneering research containing a large sample size in non-Western regions. Although the present study provided valuable results, replication of the study in other cultural contexts can test the generalizability of the findings. We tried to solve the discriminant issues of personality and general psychopathology using factor analytical techniques. We are not sure that the methods and tools used in the present study have done this well. For example, factor analytical methods are affected by the factor exploratory and rotation methods, and this can increase the instability of the results. However, future work may use tools that can better distinguish varieties of psychopathology from one another. Considering the development of transdiagnostic approaches in psychopathology, replacing symptomatology with higher-order factors proposed in recent psychopathological systems (Kotov et al., 2017; Krueger and Hobbs, 2020) may provide valuable data for mental health professionals and clinicians. We designed a short SPSQ questionnaire to measure both pseudo-cardiac symptoms and poor sleep quality. Although the reliability and validity of this scale were supported by our data, the different scoring of items adapted from three different tools caused heterogeneity in scoring. Therefore, using the same format for scoring all items and testing their validity and reliability in other communities can be useful. Future studies could use standardized measures of poor sleep quality and research instruments that cover a larger number of pseudo-cardiac symptoms. Finally, we did not exclude obese people from the study. This can be considered as an entry criterion in future studies.

## Conclusion

The different capabilities of two unique factors for the symptoms of PDs and CDs were confirmed by factor analytical methods and regression analysis techniques. Although each of the symptoms of PDs and CDs independently contributes to the manifestation of pseudo-cardiac symptoms and poor sleep quality, the CDs factor is the psychopathological core. Although this may be key data for mental health professionals and clinicians, future studies could focus on psychological symptomatology suggested by recent systems of psychopathology.

## Data availability statement

The raw data supporting the conclusions of this article will be made available by the authors, without undue reservation.

## Ethics statement

This study was approved by the ethics committee of Mind GPS Institute, Kermanshah, Iran (ID: IR.MGPSI.1400.02). The patients/participants provided their written informed consent to participate in this study.

## Author contributions

MB and SK collaboratively designed and conducted the study. SK did the search process, data collection, and data analysis. MB was prepared the first draft. SK was performed the critical review process. All authors contributed to read and approved the manuscript.

## Conflict of interest

The authors declare that the research was conducted in the absence of any commercial or financial relationships that could be construed as a potential conflict of interest.

## Publisher’s note

All claims expressed in this article are solely those of the authors and do not necessarily represent those of their affiliated organizations, or those of the publisher, the editors and the reviewers. Any product that may be evaluated in this article, or claim that may be made by its manufacturer, is not guaranteed or endorsed by the publisher.
