# Supplementary material for: Which symptoms are the psychopathological core affecting the manifestation of pseudo-cardiac symptoms and poor sleep quality in young adults? Symptoms of personality disorders versus clinical disorders
Source: Front Psychol. 2022 Dec 9;13:1011737. doi: 10.3389/fpsyg.2022.1011737 (PMC9784461; doi:10.3389/fpsyg.2022.1011737)
Supplement: Supplementary file 1 [file Table_1.DOC]

**Supplementary Table.** The mean, standard deviation, and correlations between the SCL-90 and PDQ-4 symptom scales

| Variables (Mean ± SD) | 1 | 2 | 3 | 4 | 5 | 6 | 7 | 8 | 9 | 10 | 11 | 12 | 13 | 14 | 15 | 16 | 17 | 18 | 19 |
| --- | --- | --- | --- | --- | --- | --- | --- | --- | --- | --- | --- | --- | --- | --- | --- | --- | --- | --- | --- |
| SCL-90-R |  |  |  |  |  |  |  |  |  |  |  |  |  |  |  |  |  |  |  |
| 1. Somatization (12.64 ± 9.69) | - |  |  |  |  |  |  |  |  |  |  |  |  |  |  |  |  |  |  |
| 2. OCD (13.58 ± 7.99) | .746 | - |  |  |  |  |  |  |  |  |  |  |  |  |  |  |  |  |  |
| 3. Interpersonal sensitivity (11.24 ± 7.32) | .725 | .821 | - |  |  |  |  |  |  |  |  |  |  |  |  |  |  |  |  |
| 4. Depression (15.75 ± 11.49) | .754 | .853 | .841 | - |  |  |  |  |  |  |  |  |  |  |  |  |  |  |  |
| 5. Anxiety (10.06 ± 8.28) | .818 | .809 | .835 | .850 | - |  |  |  |  |  |  |  |  |  |  |  |  |  |  |
| 6. Hostility (6.98 ± 5.07) | .661 | .698 | .770 | .717 | .758 | - |  |  |  |  |  |  |  |  |  |  |  |  |  |
| 7. Phobic anxiety (5.50 ± 5.64) | .703 | .666 | .757 | .688 | .791 | .648 | - |  |  |  |  |  |  |  |  |  |  |  |  |
| 8. Paranoid ideation (8.21 ± 5.00) | .635 | .765 | .786 | .756 | .718 | .730 | .595 | - |  |  |  |  |  |  |  |  |  |  |  |
| 9. Psychoticism (8.52 ± 7.56) | .725 | .739 | .807 | .788 | .833 | .721 | .813 | .691 | - |  |  |  |  |  |  |  |  |  |  |
| PDQ-4 |  |  |  |  |  |  |  |  |  |  |  |  |  |  |  |  |  |  |  |
| 10. Paranoid (2.97 ± 1.85) | .198 | .303 | .284 | .292 | .234 | .277 | .114 | .431 | .219 | - |  |  |  |  |  |  |  |  |  |
| 11. Schizoid (2.13 ± 1.53) | .207 | .249 | .265 | .291 | .244 | .215 | .236 | .311 | .274 | .385 | - |  |  |  |  |  |  |  |  |
| 12. Schizotypal (2.51 ± 1.86) | .279 | .282 | .321 | .295 | .319 | .277 | .300 | .370 | .350 | .431 | .512 | - |  |  |  |  |  |  |  |
| 13. Antisocial (1.66 ± 1.69) | .234 | .198 | .282 | .235 | .301 | .346 | .309 | .282 | .347 | .310 | .427 | .464 | - |  |  |  |  |  |  |
| 14. Borderline (2.64 ± 2.10) | .403 | .461 | .481 | .513 | .500 | .504 | .372 | .439 | .468 | .456 | .435 | .469 | .570 | - |  |  |  |  |  |
| 15. Narcissistic (2.82 ± 1.90) | .217 | .227 | .282 | .226 | .259 | .298 | .224 | .344 | .294 | .459 | .383 | .529 | .519 | .446 | - |  |  |  |  |
| 16. Histrionic (2.67 ± 1.79) | .208 | .258 | .302 | .254 | .274 | .283 | .253 | .321 | .276 | .385 | .220 | .393 | .478 | .472 | .535 | - |  |  |  |
| 17. Avoidant (2.01 ± 1.71) | .264 | .389 | .468 | .411 | .381 | .317 | .358 | .367 | .394 | .410 | .368 | .417 | .346 | .525 | .410 | .380 | - |  |  |
| 18. Dependent (1.87 ± 1.90) | .344 | .399 | .461 | .435 | .446 | .369 | .460 | .347 | .480 | .297 | .329 | .393 | .457 | .545 | .386 | .499 | .583 | - |  |
| 19. Obsessive-compulsive (2.92 ± 1.73) | .202 | .329 | .311 | .304 | .274 | .269 | .199 | .381 | .255 | .490 | .432 | .429 | .341 | .447 | .488 | .441 | .451 | .411 | - |

Note. All columns contain Pearson correlation coefficients (r); *p*-value for all correlations is < .001

Abbreviations_ OCD: Obsessive-compulsive disorder, SCL-90-R: Revised Form of Symptom Checklist-90, PDQ-4: Fourth Edition of the Personality Diagnostic Questionnaire, SD: standard deviation.
